# Perceived and Dispositional Hope as Health-Related Constructs: Adaptation and Validation of the Polish Version of the Perceived Hope Scale

**DOI:** 10.3390/jcm14103578

**Published:** 2025-05-20

**Authors:** Elżbieta Katarzyna Kasprzak, Karolina Mudło-Głagolska, Andreas Krafft

**Affiliations:** 1 Faculty of Psychology, Kazimierz Wielki University, 85-064 Bydgoszcz, Poland; karolina.glagolska@gmail.com; 2 Institute of Systemic Management and Public Governance, University of St. Gallen, 9000 St. Gallen, Switzerland; andreas.krafft@unisg.ch

**Keywords:** dispositional hope, perceived hope, health, personal resources

## Abstract

**Background**: The effectiveness of treatment depends on recognizing the needs and limitations of patients. Hope is a personal resource that facilitates the treatment and recovery process. Dispositional hope encompasses goal-directed action, whereas perceived hope lacks reference to specific content or behavior. This study examined which construct is more strongly related to psychological, physical, and emotional health. Perceived hope requires a new tool for measurement. Adaptation to the Polish cultural context is the second goal of this research. **Methods**: Data were collected in the international online study Barometer of Hope (n = 1608). Adult participants completed the PSH, ADHS, and a battery of self-report questionnaires assessing several key well-being outcomes. **Results**: Perceived hope appears to be a more salient construct related to psychological health than dispositional hope, although both aspects of hope demonstrate similar associations with physical health. Confirmatory factor analysis (CFA) supported the hypothesized one-factor structure of the Polish version of the Perceived Hope Scale (PHS-PL), indicating high internal consistency as well as strong convergent and discriminant validity. The PHS-PL showed positive correlations with optimism, life satisfaction, happiness, positive affect, and dispositional hope, and negative correlations with depression/anxiety, loneliness, and negative affect. Additionally, perceived hope was negatively associated with the likelihood of a crisis scenario and positively associated with the likelihood of a flourishing scenario. **Conclusions**: Our findings confirm that hope is a health-enhancing resource. The PHS is a simple, short, culturally universal method that directly measures hope and can also be successfully used by non-psychologists, such as nurses, physicians, and caregivers.

## 1. Introduction

The effectiveness of medical and psychological care depends on recognizing patients’ needs and limitations and maintaining a holistic view of their social situation, lifestyle, and cognitive and emotional functioning [1]. Patients with comorbidities, those with intellectual disabilities or mental illness, and elderly individuals with symptoms of dementia require different treatment plans, as they often experience symptoms of anxiety or depression that hinder recovery [2]. The selection of appropriate diagnostic and treatment procedures should consider this emotional factor.

This approach aligns with the principles of positive psychology, which emphasize personal resources as protective factors against disorders and diseases, thereby improving prospects for recovery and future well-being. According to Seligman and Csikszentmihalyi [3], the three pillars of personal resources that can strengthen treatment outcomes include positive life experiences, positive individual traits, and a supportive healthcare environment. The first two subjective factors relate to psychological capital [4], encompassing hope, efficacy, resilience, and optimism (HERO). Although optimism is a recognized factor in recovery, particularly in cancer, researchers have increasingly highlighted the benefits of hope in coping with various diseases in recent years, confirming that hope is a universal resource for managing a wider range of health conditions [5]. The usefulness of hope in dealing with many illnesses relates to its emotional and biological aspects. Hope alleviates sadness and anxiety, redirecting immunological processes from reducing tension to combating cancer, for example [6].

A review of studies confirms the therapeutic role of hope in treating chronic pain and chronic illnesses [7]. Hope is relevant in health challenges like cancer and chronic pain [6,8], spinal cord injuries [9], and visual impairment [10]. Hopeful individuals are more likely to engage in pro-health behaviors and avoid health-risk behaviors [11,12], which improves the effectiveness of therapy and aids recovery [6].

In psychology, hope is understood as the desire for a particular good, the belief in its attainability, and the trust in the availability of resources to cope with and overcome obstacles and difficulties [13].

Snyder emphasizes the cognitive nature of hope, which is expressed in the agency to formulate and achieve goals and the self-assessed ability to generate solutions for their attainment [14]. For other authors, hope involves trust and a desire that directs human actions, thoughts (even passive waiting for favorable circumstances), and emotions towards outcomes and events significant to the individual [15].

Several researchers focus on the emotional nature of hope, which can be more of an experience than an action or expectation [16]. This means that hope, as an emotion, is less controlled, more spontaneous, biologically conditioned, and varies in intensity and valence [6]. Hope is experienced as hopefulness, serves developmental purposes, broadens the field of perception, and helps build personal resources and resilience, contributing to human mastery [17,18].

Cognitive and emotional processes can reinforce each other, promoting engagement and perseverance or maintaining hope in dire situations. The motivational function of hope, based on wishes, trust [13], willpower, and waypower, supports goal achievement [14,16] or generally the fulfilment of hope [13]. The motivational nature of hope also reveals itself in its stability; people are full of hope “despite everything.” This is also because robust hope is resilient to the psychological costs of disappointment since, by nature, it is uncertain. Therefore, even failure does not weaken it, which can occur with strong expectations [15,16].

Hope encompasses an element of uncertainty [15,18] or irrationality when an individual persists in their object of hope despite contextual difficulties, even if only passively [15]. The mechanism of hope is based on the belief that problems are temporary and can improve, but also on the desire for a good outcome, even with little or no chance of its occurrence [19]. Persisting in hope “despite everything” demonstrates that hope, on the one hand, drives action. At the same time, on the other hand, it is a patient’s expectation of good (sometimes in line with spiritual faith), as it offers the possibility of a better life with the disease, going beyond mere survival [8]. Active enhancement of hope throughout diagnosis, treatment, and follow-up care is possible [6] and should be incorporated into a personalized treatment plan. Hope as an element of customized treatment may prove valuable for patients who feel desperate, helpless, passive, or face additional pain or cognitive burden. Patients with difficulties in generating action plans and perceiving themselves as agents may have greater challenges in practicing goal-based hope; thus, an opportunity for them is to cultivate non-specific hope, which is expressed in the wish that the disease symptoms will subside or that life with the disease will improve regardless of the circumstances and chances for improvement [8]. The function of non-specific hope can be fulfilled by the construct of perceived hope [20]. This conception of hope omits references to the content, spheres, and essence of hope and instead refers to the personal understanding of hope. Hope, as perceived by the general public, is treated as a cognitively affective–transcendent experience that does not necessarily relate to a goal or the individual’s agency. Moreover, these factors do not serve as criteria for recognizing or not recognizing someone’s experience as hope. Empirical support has been found for such a defined understanding of hope. Results of free associations regarding hope confirm the assumption of a universal understanding of hope among people from different cultures and show that this understanding is similar across cultures and consistent with earlier scientific findings [21]. Perceived hope [20] describes hope only in formal dimensions: the level of hope and its fulfillment, as a counterbalance to anxiety (in line with the duality of emotions: hope–anxiety or depression [2,8,13]); and the effects of hope, especially in difficult situations. Perceived hope provides a new, broader approach but also complements the idea of hope omitted in Snyder’s most widespread model of dispositional hope [14].

The distinction between perceived hope and dispositional hope concerns several issues. Dispositional hope includes two components: willpower and waypower, which support the formulation of goals and ways of achieving them. Dispositional hope is a mainly cognitive construct because it is based on self-trust and belief in the possibility of achieving the goal. Perceived hope, on the other hand, is an emotional–cognitive–transcendent construct, encompassing only the formal aspects of hope (extent of fulfillment, strength, effects, and lack of fear) and referring to the subjective experience/feeling of hope. The dispositional hope model directly relates to the agency and purposefulness of activity, which is closely associated with self-confidence, overlooking individual experiences of hope and its “passive” action [8,15,19,20]. This gap is filled by the construct of perceived hope, which is less strongly associated with activity and self-efficacy and more strongly associated with emotional markers. Perceived hope, in comparison to dispositional hope, is more strongly associated with both positive and negative emotions [22], spiritual beliefs and religious faith, depression and anxiety, and positive mental health [20], as well as the willingness to help others and gratitude, but less strongly with self-efficacy and resilience [20]. At a similar level, both natures of hope are associated with generativity, [12,20] engagement in passion, and volunteering [22]. The strength of the relationship between hope and other constructs results from the different definitions of hope as a mainly cognitive or formal–emotional construct. We do not know exactly why and when the emotional, cognitive, and transcendent aspects of hope become primary for a person but we assume that all these aspects are interconnected. We believe that the development of this agentive hope or perceived hope occurs simultaneously because it concerns the same construct, that it is confirmed by the high correlation coefficients between these levels of hope.

### Current Study

The primary objective of this research is to demonstrate the explanatory power of hope concerning various health indicators, depending on the model of hope.

Previous studies investigating the role of hope in the healing process have most recently utilized the Snyder and Herth Hope models [6]. Both possess strong motivational and cognitive components linked to action and purpose. The Herth Hope Index also evaluates other constructs beyond hope, including loneliness, positive memories, and religiosity, which may blur the lines between hope and other constructs [6]. Measuring the cognitive–motivational nature of hope does not always enable an accurate assessment. Seniors with dementia or individuals with cognitive challenges face objective or subjective limitations in planning actions and perceiving themselves as agents. However, they may experience hope as a diffuse, non-specific emotion that does not necessitate planning actions or striving for a goal. Furthermore, healthy individuals experience hope based on their feelings, whether they associate it with a specific goal or intend to take action toward it.

The above considerations enable us to propose a hypothesis (H1) that perceived hope, more effectively than dispositional hope, accounts for psychological, physical, and emotional health.

The second goal of this research is to present a perceived hope scale in the Polish language version. This simple, short, quantitative, culturally universal, and direct measure of hope [20] can be used by non-psychologists to assess the strength of patients’ hope.

The scale has been validated in German [20], English [23], Czech [24], and Portuguese [25], indicating the growing interest in perceived hope and enabling comparisons of this construct in a cultural context. The PHS demonstrated good or excellent reliability coefficients and structural and criterion validity in all validations. We, therefore, assume (H2) that the Polish version of the PHS has a one-factor structure and achieves satisfactory psychometric parameters, comparable to previous adaptations.

## 2. Method

### 2.1. Procedure and Materials

The study employed data from the Polish editions of a yearly cross-sectional international online survey entitled Hope Barometer. The Hope Barometer study is conducted in collaboration with national media outlets, including press and television, where invitations for participation are posted. All interested adults from the general population are invited to take part in the study. The study was anonymous and voluntary, and all participants provided written informed consent for use of their data. The sample is further expanded through snowball sampling. The study was conducted according to the ethical principles of the Declaration of Helsinki. Ethical approval for this research was granted by the Kazimierz Wielki University Ethics Committee (8 October 2015; 10 October 2017). Data collected in the 2017 and 2019 editions were used to verify the hypothesis of stronger associations between health and other quality of life indicators with perceived hope than dispositional hope, and to analyze the convergent and discriminant validity of the perceived hope scale (PHS). The criterion variables included physical health, mental health, optimism, life satisfaction, happiness, emotional experiences, dispositional hope, symptoms of depression and anxiety, and feelings of loneliness. Data from the 2015 and 2016 editions were used to evaluate PHS’s factorial structure (CFA) and internal consistency. The external criterion for the scale’s validity was a question regarding the likelihood of long-term societal future scenarios: social crisis and social flourishing.

The Perceived Hope Scale consists of six items. Respondents rated these items on a 6-point Likert scale, ranging from 0 (strongly disagree) to 5 (strongly agree). A high score on the scale indicates a high level of perceived hope. The English version of the PHS was translated into Polish by three independent translators proficient in both languages, resulting in a cohesive Polish rendition of the questionnaire. A Polish language expert reviewed all statements to ensure clarity and grammatical correctness. Subsequently, the questionnaire was translated back into English, and this back translation was compared with the original version of the PHS. All statements were found to be either identical or comparable, confirming the consistency of the Polish-language statements with the English version [23]. This set of statements was used in the validation studies.

Health was measured using two items: one regarding self-assessment of physical health and the other concerning self-assessment of mental and emotional health. Responses were marked on a scale ranging from 1 (I am seriously ill) to 6 (my health is excellent). The questions were drawn from the European Study on Adult Well-being.

Optimism was measured using the LOT-R scale by Scheier et al. [26] in the Polish adaptation by Juczyński [27], which consists of six diagnostic items. Participants responded on a 6-point scale, ranging from 1 (strongly disagree) to 6 (strongly agree). Three items are positively worded, while three are negatively worded. A higher score indicates a higher level of optimism. Similar to the Czech and Portuguese adaptations [24,25], separate scores for optimism (three positively worded statements) and pessimism (three negatively worded statements) were calculated for these validation analyses.

Dispositional hope was measured using Snyder’s ADHS [28], which consists of eight items; four cover the agency subscale, while the other four cover the pathway subscale. Participants responded on a 6-point scale (from 0 to 5), meaning each component could yield between 0 and 20 points, with a higher score indicating a higher level of hope. This study used a Polish adaptation of this scale [29].

Life satisfaction was measured using the Satisfaction with Life Scale by Diener et al. [30], adapted into Polish by Juczyński [27]. This scale consists of five statements rated on a 7-point scale (from 1 to 7), where a higher score indicates greater satisfaction with life. Subjective happiness was assessed using the Subjective Happiness Scale [31], which consists of four items. Participants marked their responses on a 7-point scale indicating the extent of their agreement with each statement. A higher score reflects a greater sense of happiness.

Experienced emotions were measured using the Scale of Positive and Negative Experience [32], comprising six items related to positive emotions and six associated with negative emotions. Results from both scales indicate the frequency of positive and negative emotions experienced over the past four weeks. Participants responded on a scale from 1 (rarely or never) to 5 (often or always). A higher score reflects a greater frequency of both positive and negative affect.

Depression and anxiety were measured using the ultra-brief Patient Health Questionnaire for Depression and Anxiety (PHQ-4) [33], which consists of four items related to emotions, with participants indicating on a scale from 0 to 3 how often they had experienced these emotions in the past two weeks.

The loneliness scale from the Adult Toolbox Social Relationship Scale [34] was utilized as the final criterion. The loneliness subscale comprises five items related to feelings of loneliness and abandonment, with participants indicating on a scale from 1—never to 5—always the frequency with which they had experienced such feelings in the past year. A higher score reflects a greater frequency of loneliness.

As an external criterion, participants evaluated the likelihood of two scenarios describing the future from the distant perspective of 2040 [35]. The crisis scenario, “More population, environmental destruction, new diseases and ethnic and regional conflicts mean the world is heading for a bad time of crisis and trouble,” and the flourishing scenario, “By continuing on its current path of economic and technological development, humanity will overcome the obstacles it faces and enter a new age of sustainability, peace and prosperity,” were rated on a probability scale from 1—Very unlikely to 6—Very likely.

### 2.2. Participants

Psychometric validation was performed on four samples. Sample 1 includes participants from two measurements in 2015 and 2016. Sample 4 is a merged sample of Samples 2 and 3, as participants completed the same questionnaires regarding perceived hope, dispositional hope, physical and mental health, and feelings of anxiety and depression. The purpose, measures used in the study, and detailed sociodemographic characteristics of the sample are presented in Table 1.

### 2.3. Data Analysis

To test hypothesis 1, linear regression models were constructed, and the regression coefficients of perceived and dispositional hope as explanatory factors for physical and mental health were compared. Hypothesis 2 was tested using several methods to assess the validity and reliability of the PHS. The factor model of the PHS was tested by CFA, using maximum likelihood estimation with robust standard errors. The fit of the factor model was assessed based on the following common fit indexes [36]: the ratio of Chi^2^ to degrees of freedom (Chi^2^/df), root mean square error of approximation (RMSEA), standardized root mean square residual (SRMR), comparative fit index (CFI), and Tucker–Lewis index (TLI). Chi^2^/df is considered acceptable when it is less than or equal to 5 [37]. RMSEA and SRMR values below 0.08, along with CFI and TLI values greater than 0.90, indicate an acceptable fit, while RMSEA values below 0.06 and CFI and TLI values greater than 0.95 indicate an excellent fit [36]. Measurement invariance analysis was performed in configural, metric, and scalar in merged samples. In testing metric and scalar invariance, ΔCFI of <0.01 and ΔRMSEA of <0.015 indicate invariance [38].

McDonald’s omega values (ω) and Cronbach’s alpha coefficients (α), along with their 95% confidence intervals, were calculated. For these coefficients, values ≥ 0.70 were judged acceptable, ≥0.80 good, and ≥0.90 excellent [39].

We calculated Pearson correlations between PHS scores and physical and psychological health, LOT-R (optimism), ADHS (agency and pathways), SWLS (life satisfaction), SPANE-P (positive emotion), and the flourishing scenario to assess convergent validity. We also evaluated correlations between PHS scores and SPANE-N (negative emotion), ATSRS (loneliness), PHQ-4 scores (anxiety and depression symptoms), and the crisis scenario to assess the discriminant validity of the PHS. The above analyses were conducted using JASP version 0.16.0.0 for Windows [40]

## 3. Results

### 3.1. Descriptive Statistics and Reliability of Tools

Table 2 presents descriptive statistics for all study variables. Across all samples, variable skewness scores ranged from 0.12 to −1.57, while kurtosis scores ranged from −0.07 to 3.76 (the latter for LOT-R), indicating that the study variables were reasonably normally distributed. The distribution shape was considered close to normal when skewness < 3 and kurtosis < 8 [41]. As shown in Table 2, the PHS total score exhibited good internal consistency reliability (α and ω = 0.91).

We checked the measurement invariance of the PHS-PL across four separate test groups: the 2015 and 2016 samples included in sample 1, and the 2017 and 2019 samples that were merged into sample 4. In each group, the proposed one-factor model demonstrated an excellent fit to the data. The analyses indicated that the one-factor model achieved configural, metric, and scalar invariance across the different groups (Table 3). This means that the PHS-PL reveals strong invariance, which allows for the use of samples 1 and 4 in further analyses: CFA and the correlation of PHS with other variables.

### 3.2. Health and Perceived Hope Versus Dispositional Hope

The results of linear regression confirmed that perceived hope explains psychological and emotional health better than dispositional hope (Table 4). However, to a similar extent close to zero, both types of hope explain physical health.

As expected, the regression analysis results support hypothesis H1, although it pertains only to emotional and psychological health. Similarly, both aspects of hope explain physical health to a comparable degree.

### 3.3. Measurement Invariance of the PHS in Three Language Groups

The original version of the PSH was prepared in parallel in two languages: German and English. To analyze the linguistic invariance data from three language versions—German (n = 2406), English (n = 228), and Polish (n = 937)—from the Hope Barometer project conducted in 2015 and 2016, the indices of metric and scalar invariance across the three language samples presented in Table 5 confirm the linguistic invariance of the PHS.

### 3.4. Factor Structure of PHS

We tested a theoretically informed one-factor model of the PHS, where all six items were specified to load onto a general perceived hope factor. We used survey data from 2015 to 16 (sample 1; n = 937).

The fit indices obtained indicate a good model fit to the data (Chi^2^ = 33.568, *df* = 9, Chi^2^/*df* = 3.73, *p* < 0.001, RMSEA (90% CI) = 0.054 (.035–074), SRMR = 0.018, CFI = 0.993, TLI = 0.989). Factor loadings (all *p* < 0.001) for items within the entire tested model ranged from 0.68 (for the first item) to 0.91 (for the fifth item) (see Table 6). In summary, empirical support was found for the one-factor structure of PHS-PL. As expected, CFA results support hypothesis H2.

### 3.5. Convergent and Discriminant Validity

To establish the convergent and discriminant validity of PHS-PL, several criterion variables associated with hope were selected. These criterion variables included physical health and mental health, optimism, life satisfaction, happiness, emotional experiences, dispositional hope, symptoms of depression and anxiety, feelings of loneliness, and consideration of future societal scenarios from a distant time perspective (cf. flourishing, crisis scenarios). Detailed results are presented in Table 7.

Positive relationships were observed between perceived hope and indicators of positive functioning. Strong positive associations were demonstrated between perceived hope and agency (dispositional hope subscale), life satisfaction, subjective happiness, and positive affect. A weak positive association was noted between physical health and hope (regardless of the measurement method). A weak negative relationship was observed between perceived hope and the crisis scenario. Additionally, a moderate negative relationship was confirmed between perceived hope and loneliness, alongside a strong negative relationship with symptoms of anxiety and depression, as well as negative affect. ADHS correlates less strongly than PHS-PL with variables related to felt emotions (optimism, anxiety/depression, crisis scenario) and psychological health. As expected, the correlation coefficients support hypothesis H2 regarding convergent and discriminant validity and additionally support hypothesis H1 regarding the relationship between health and the two types of hope.

## 4. Discussion

The results of our research confirmed that hope can be a good predictor of health (H1). According to the hypothesis, perceived hope is a better predictor of psychological and emotional health than dispositional hope, which is consistent with previous results. Several mechanisms explain the relationship between hope and psychological health. Hope for achieving a goal motivates action, strengthens self-assessment as an agent, and reduces negative emotions [20], thereby improving psychological and mental health [5,6]. The experience of hope enhances health by blocking negative information and its consequences [18,42], while redirecting attention to positive self-information [20]. In addition, the feeling of hope encourages individuals to choose more difficult goals, which builds competence and self-esteem, subsequently strengthening hope. Moreover, the feeling of hope, as an expectation of positive outcomes and goodness in general in the face of difficulties, fosters proactive behaviors [9,11]. All these mechanisms pertain to multifactorial determinants of health, with some emphasizing the regulatory role of hope in activating other resources that are more directly related to health than hope itself. Research evidence indicates that hope is a stronger predictor of health through the mediating and moderating role of one’s own or other resources, such as resilience [43], stress, depression, exposure to negative events [14,44], health behaviors [6,12], and age [45]. The hope mechanism works by activating personal resources. A hopeful person believes that good things will happen, that obstacles will be overcome, and that the planned goal will be achieved. These positive beliefs and the accompanying positive emotions of expectation, readiness, and openness strengthen the motivation to act in order to make one’s hopes come true. The motivation of a sick person is manifested in undertaking physical activity and following a healthy diet and medical recommendations, and such behavior directly supports the healing process. On the other hand, a person struggling with a crisis or loss who hopes for a successful problem solution experiences positive emotions, which strengthen self-confidence and a sense of agency. These emotions and beliefs directly strengthen health and protect against stress or burnout. A sense of agency and positive affect are activated by the prospect of success that hope gives. These mechanisms and results suggest the existence of an overarching factor, such as psychological capital, which is effective in coping with difficulties and illness when all HERO components collaborate and support one another [4].

However, our results do not support the conclusion that physical health is more strongly related to perceived hope than to dispositional hope. Both types of hope, although significant predictors of physical health, explain it only to a small extent. This limited explanatory value of hope stems from the sample characteristics. Relatively young respondents (M = ~36 years) are generally physically and mentally healthy, as confirmed by their average health scores. For such individuals, hope that provides strength, distracts from anxiety, or motivates action is less essential. Routine care for physical health (e.g., seeking medical advice, engaging in health-promoting behaviors) does not necessitate an additional personal resource—hope.

Perhaps perceived hope comes into play when a person feels helpless and desperate, when they are surprised by a diagnosis, a medical prognosis, or by the discomforts of treatment. This explanation is shared by researchers of cancer patients [6,12], people with chronic diseases and pain, or those experiencing the burden of therapy [8]. It is possible that effects mediated by mental health (e.g., lack of feelings such as depression, anxiety, and sadness) would show a stronger explanatory power of hope in relation to physical health.

We have much evidence showing a positive relationship between hope and well-being among young people (e.g., success at school, satisfying relationships, and achievements) [46]. However, the direct relationship between hope and health is weak [43]. These results indicate an indirect relationship between hope and health. Hope indirectly facilitates the healing and recovery process [6], e.g., by leading a healthy lifestyle, enjoying social support, or following medical recommendations.

Nevertheless, hope always works most strongly in times of crisis and despair. Hope, as a feeling, does not need an object or supporting context to relieve tension and protect against illness. It activates cognitive mechanisms, such as redirecting attention, reformulating the problem, or providing new meaning, which helps to overcome difficult emotions [42]. Strengthening, or even diagnosing, hope seems useful in planning personalized healthcare, especially for patients with hope deficits.

The second research goal was also achieved (H2). The fit indices indicate a good fit of the model to the data that supports the hypothesized 1-factor model of the Perceived Hope Scale (PHS-PL), where all six items load onto a general perceived hope factor. This finding lends empirical support to the theoretical model of perceived hope, demonstrating that the PHS-PL is a reliable and valid measure for assessing this construct.

The convergent and discriminant validity findings align with previous research in this area. Positive and at least moderate associations were confirmed between perceived hope and various positive functioning indicators: positive affect, life satisfaction, and subjective happiness. The strength and directions of the revealed correlations are similar to those found in the German [20] and South African [23] validations. Optimism in the Polish sample is correlated with PHS-PL at a level of 0.71, similar to the high correlations observed in Czech [24] and South African studies [23]. Positive relationships between perceived hope and self-rated physical and psychological health (r = 0.25 and r = 0.51, respectively) are consistent with the hypothesis. Discriminant validity has also been demonstrated. The correlations between the PHS-PL and depression and anxiety symptoms (r = −0.51) also mirror previous findings, such as those in the Czech adaptation of PHS, where this relationship was −0.55 [24], and in the German-speaking sample, where it was −0.51 [20]. The correlation between PHS-PL and negative affect (r = −0.57) supports these results. Our findings regarding the relationships of PHS-PL with negative emotions, feelings of anxiety–depression, and loneliness are consistent with earlier validations [20,24,25] and global studies [16,44].

Our results confirm that the PHS-PL is a new way of measuring hope as it is experienced and understood by the general public in Poland. It complements existing hope measurement tools. The scale is brief, encouraging motivation for completion, and it is understandable for the average respondent, building trust that it measures what people associate with hope. We believe it can be used to assess hope in clinical groups, such as individuals with low literacy and mild cognitive impairments, as well as in healthy groups in screening studies, including older children, hospitalized individuals, or those experiencing life transitions, toward flourishing.

Further support for the validity of PHS-PL comes from the estimated likelihood of future scenarios: crisis or flourishing in the macro-social dimension. The negative relationship between perceived hope and the crisis scenario indicates that individuals with higher levels of hope see a pessimistic view of the future as less likely. Conversely, hopeful people view an optimistic vision of the future as more likely. These beliefs—negative towards crisis and positive towards the flourishing of humanity and the future of civilization—are consistent with the negative correlation between dispositional and perceived hope with loneliness, as well as a strongly negative correlation with symptoms of anxiety, depression, and negative affect. This underscores the protective role of hope against feelings of loneliness and psychological distress.

The consistent direction and strength of the relationship between PHS-PL and ADHS confirm that both constructs measure hope. Compared to ADHS, PHS-PL shows stronger associations with psychological health, optimism, anxiety–depression feelings, and the crisis scenario (trend), which more strongly support the emotional aspect of perceived rather than dispositional hope. Similar differences have been revealed in Czech [24] and Swiss (German-speaking) studies [20], confirming that perceived hope is a universal health factor better explained by PHS than by ADHS. In summary, the results obtained using different methods of testing the validity of the PHS-PL scale confirm the good psychometric parameters of the scale.

### Limitations of the Research and Future Directions

These samples do not strictly represent the Polish population but focus on individuals who are internet-literate and have access to the internet. Moreover, these samples are not homogeneous concerning sociodemographic variables. The Polish sample is relatively homogeneous regarding religious affiliation, thus limiting generalizations about hope among Catholics. Two social phenomena—progressive secularization and immigration to Poland, albeit slow—are prompting the need for future research to include individuals of other faiths or those without religious affiliation. Despite these limitations, we believe that the results obtained are sufficiently generalizable and well-balanced.

As a direction for future research, it is worth considering testing mediation and moderation models. A role of mediator or moderator may be played by optimism, resilience, or a sense of self-efficacy, i.e., psychological capital resources. Certainly, the predictive value of perceived and dispositional hope in predicting health should be tested, firstly, in longitudinal studies and, secondly, in clinical groups; and among people with somatic or mental illnesses and among patients with chronic or terminal diseases.

## 5. Conclusions

Our findings confirm that hope is a health-enhancing resource. This is particularly significant because this positive relationship is observed in a sample of relatively healthy individuals. Mental health is better explained by perceived hope than by dispositional hope, which justifies adopting this framework to assess hope in certain clinical groups.

Similar to previous PHS validations, its Polish cultural version supports that perceived hope may have a direct or indirect beneficial connection with various aspects of individuals’ functioning and emotional well-being and protect against anxiety, depression, and loneliness. The Polish version of the PHS-PL, whose psychometric properties we presented in this article, is available at the Appendix A.

## Figures and Tables

**Table 1:** The aims of the study and sociodemographic characteristics of the study samples.

	Sample 1 (2015–2016) *n* (%)	Sample 2 (2017) *n* (%)	Sample 3 (2019) *n* (%)	Sample 4 (2017, 2019) Total, *n* (%)
Purpose and applied measures	Factorial validity	Convergent and discriminant validity: (SWLS, SHS, SPANE, LOT-R)	Convergent and discriminant validity: (ATSRS, crisis and flourishing scenario)	Hope as a predictor of health; convergent and discriminant validity: (physical and psychological health, ADHS, PHQ-4)
Total	937 (100)	190 (100)	481 (100)	671 (100)
Gender				
Male	288 (35)	78 (41.1)	140 (29.1)	218 (32.5)
Female	649 (65)	112 (58.9)	341 (70.9)	453 (67.5)
	2015 year			
Age (years)				
18–29	6 (1.12)	M = 45.1 SD = 15.6 (min = 19, max = 89)	M = 31.6 SD = 10.8 (min = 18, max = 82)	M = 35.6 SD = 13.8 (min = 18, max = 89)
30–39	261 (48.78)			
40–49	106 (19.8)			
50–59	61 (11.40)			
60–69	60 (11.21)			
70+	41 (7.7)			
	2016 year			
	M = 39.4 SD = 14.4 (min = 18, max = 81)			
Education				
Not finished and primary	23 (2.3)	8 (4.3)	4 (0.8)	2 (1.7)
Secondary and post-secondary	362 (36.2)	63 (33.1)	207 (42.9)	270 (40.3)
Higher education	552 (55.2)	119 (62)	270 (56.1)	389 (47.9)
Family status				
Living with parents	165 (15.5)	15 (7.9)	85 (17.7)	100 (14.3)
Single/unmarried	198 (19.8)	27 (14.2)	71 (14.8)	98 (14)
Living in partnership	127 (12.7)	29 (15.2)	144 (29.8)	173 (24.7)
Married	397 (39.7)	101 (53.2)	166 (34.5)	267 (38.1)
Divorced/separated	31 (3.1)	10 (5.3)	14 (2.9)	24 (3.4)
Widowed	19 (1.9)	8 (4.2)	1 (0.3)	9 (1.3)

**Table 2:** Descriptive statistics, Cronbach’s alpha (α), and McDonald’s omega values (ω) with 95% confidence intervals (CI) for the study tools.

	α (95 CI)	ω (95 CI)	*N*	*M*	*SD*
sample 1					
PHS	0.91 (0.90–0.92)	0.91 (0.90–0.92)	937	3.08	1.55
Samples 4 (*N* = 671), 2 (*N* = 190), and 3(*N* = 481)					
PHS	0.91 (0.90–0.92)	0.91 (0.90–0.92)	671	3.21	1.06
Physical health	–	–	671	4.49	0.95
Psychological health	–	–	671	4.68	1.05
LOT-R	0.86 (0.84–0.87)	0.87 (0.84–0.89)	671	3.67	0.62
ATSRS	0.92 (0.91–0.93)	0.92 (0.91–0.93)	671	1.72	1.34
ADHS total	0.90 (0.88–0.91)	0.90 (0.89–0.91)	671	28.4	6.98
SWLS	0.91 (0.89–0.92)	0.91 (0.88–0.93)	190	3.89	1.40
SHS	0.88 (0.84–0.90)	0.88 (0.85–0.91)	190	4.35	1.45
SPANE-P	0.94 (0.92–0.95)	0.94 (0.92–0.95)	190	3.40	0.86
SPANE-N	0.92 (0.91–0.94)	0.93 (0.91–0.94)	190	2.72	0.96
PHQ-4	0.90 (0.89–0.91)	0.90 (0.88–0.92)	481	1.01	0.80
Crisis scenario	–	–	481	4.35	1.15
Flourishing scenario	–	–	481	3.23	1.33

PHS = The Perceived Hope Scale, LOT-R = The Life Orientation Test-Revised, ADHS = The Adult Dispositional Hope Scale, SWLS = The Satisfaction with Life Scale, SHS = The Subjective Happiness Scale, SPANE = The Scale of Positive and Negative Experience, ATSRS = The Adult Toolbox Social Relationship Scale.

**Table 3:** Measurement invariance analysis of the PHS across samples.

Model	Chi^2^/*df*	CFI	ΔCFI	RMSEA	ΔRMSEA
Sample 1					
Configural	48.402/18	0.992	-	0.060	-
Metric	53.517/23	0.991	0.001	0.053	0.007
Scalar	62.875/28	0.990	0.001	0.052	0.001
Sample 4					
Configural	40.870/18	0.999	-	0.062	-
Metric	46.539/23	0.999	0	0.055	0.007
Scalar	72.390/46	0.999	0	0.041	0.014

Note: RMSEA = root mean square error of approximation, CFI = comparative fit index.

**Table 4:** Linear regression, results for health, hope as predictor, separate models for each aspect of hope.

Predictors	Perceived Hope				Dispositional Hope			
	B	95% CL of B	SE	β	B	95% CL of B	SE	β
Psychological health	0.53	0.45–0.61	0.04	0.51 **	0.5	0.40–0.60	0.05	0.17 **
	R^2^ = 0.26				R^2^ = 0.17			
Physical health	0.22	0.15–0.30	0.04	0.25 **	0.25	0.15–0.34	0.05	0.23 **
	R^2^ = 0.06				R^2^ = 0.05			
Depression/anxiety (Emotional health)	−0.41	−0.48–0.35	0.03	−0.51 **	−0.41	−0.49–0.34	0.04	−0.44 **
	R^2^ = 0.26				R^2^ = 0.19			

Note: ** *p* < 0.001; Cl Confidence interval.

**Table 5:** Measurement invariance analysis of the PHS across three language samples.

Model	Chi^2^/*df*	CFI	ΔCFI	RMSEA	ΔRMSEA
Configural	116.355/27	0.999	-	0.053	-
Metric	233.759/37	0.998	0.001	0.067	0.014
Scalar	355.781/83	0.997	0.001	0.053	0.014

Note: RMSEA = root mean square error of approximation, CFI = comparative fit index.

**Table 6:** Descriptive statistics of the PHS statements and standardized factor loadings from confirmatory factor analysis.

PHS Statement	*M*	*SD*	Skewness	Kurtosis	Factor Loadings
In my life, hope outweighs anxiety.	3.10	1.36	−0.53	−0.51	0.68
2. My hopes are usually fulfilled.	2.83	1.24	−0.43	0.08	0.69
3. I feel hopeful.	3.21	1.31	−0.73	−0.02	0.87
4. Hope improves the quality of my life.	3.12	1.32	−0.55	−0.29	0.77
5. I am hopeful with regard to my life.	3.22	1.34	−0.75	−0.07	0.91
6. Even in difficult times, I am able to remain hopeful.	3.20	1.29	−0.68	−0.09	0.81

**Table 7:** Bivariate correlations between PHS and ADHS with criterion variables, and correlation comparisons (PHS and ADHS).

Variables and Measurement Tools	Perceived Hope (PHS-PL)	Dispositional Hope (ADHS)	*z*	*p*
Physical health	0.25 ***	0.23 ***	0.388	0.349
Psychological health	0.51 ***	0.41 ***	2.323	0.010
Optimism (LOT-R)	0.73 ***	0.62 ***	1.970	0.024
Pessimism (LOT-R)	−0.50 ***	0.45 ***	−0.625	0.266
Optimism–pessimism (LOT-R)	0.71 ***	0.62 ***	1.568	0.058
Satisfaction with life (SWLS)	0.60 ***	0.69 ***	−1.497	0.067
Subjective happiness (SHS)	0.59 ***	0.65 ***	−0.944	0.173
Positive emotions (SPANE-P)	0.68 ***	0.65 ***	0.52	0.301
Negative emotions (SPANE-N)	−0.57 ***	−0.54 ***	−0.419	0.337
Anxiety/depression (PHQ-4)	−0.51 ***	−0.43 ***	−1.879	0.030
Loneliness (ATSRS)	−0.42 ***	−0.36 ***	−1.095	0.137
Crisis scenario	−0.23 ***	−0.13 ***	−1.599	0.055
Flourishing scenario	0.26 ***	0.21 ***	0.818	0.207
Dispositional hope–total (ADHS)	0.58 ***			

*** *p* < 0.001.

## Data Availability

The data that support the findings of this study are available from the corresponding author [E.K.], on request.

## Appendix A

Polish version of Perceived Hope Scale—English version of Perceived Hope Scale

Skala Spostrzeganej Nadziei (PHS-PL)—Perceived Hope Scale (PHS-EN)
Jak odnosisz się do poniższych stwierdzeń?—How do the following statements apply to your personality?
W moim życiu nadzieja przeważa nad lękiem—In my life hope outweighs anxiety
Moje nadzieje zazwyczaj są spełnione—My hopes are usually fulfilled
Jestem pełen/pełna nadziei—I feel hopeful
Nadzieja podnosi jakość mojego życia—Hope improves the quality of my life
Jestem pełen/pełna nadziei co do mojego życia—I am hopeful with regard to my life
Nawet w trudnych czasach potrafię zachować nadzieję—Even in difficult times I am able to remain hopeful
Odpowiedzi: 0-zdecydowanie nie zgadzam się; 1-nie zgadzam się; 2-trochę się nie zgadzam; 3-trochę zgadzam się; 4-zgadzam się; 5-zdecydowanie zgadzam się—Answers: 0-strongly disagree; 1-disagree; 2-somewhat disagree; 3-somewhat agree; 4-agree; 5-strongly agree
